# Building One-Shot Semi-Supervised (BOSS) Learning Up to Fully Supervised Performance

**DOI:** 10.3389/frai.2022.880729

**Published:** 2022-06-02

**Authors:** Leslie N. Smith, Adam Conovaloff

**Affiliations:** ^1^US Naval Research Laboratory, Washington, DC, United States; ^2^NRC Postdoctoral Fellow, US Naval Research Laboratory, Washington, DC, United States

**Keywords:** one-shot learning, semi-supervised learning, image classification, deep learning, computer vision

## Abstract

Reaching the performance of fully supervised learning with unlabeled data and only labeling one sample per class might be ideal for deep learning applications. We demonstrate for the first time the potential for building one-shot semi-supervised (BOSS) learning on CIFAR-10 and SVHN up to attain test accuracies that are comparable to fully supervised learning. Our method combines class prototype refining, class balancing, and self-training. A good prototype choice is essential and we propose a technique for obtaining iconic examples. In addition, we demonstrate that class balancing methods substantially improve accuracy results in semi-supervised learning to levels that allow self-training to reach the level of fully supervised learning performance. Our experiments demonstrate the value with computing and analyzing test accuracies for every class, rather than only a total test accuracy. We show that our BOSS methodology can obtain total test accuracies with CIFAR-10 images and only one labeled sample per class up to 95% (compared to 94.5% for fully supervised). Similarly, the SVHN images obtains test accuracies of 97.8%, compared to 98.27% for fully supervised. Rigorous empirical evaluations provide evidence that labeling large datasets is not necessary for training deep neural networks. Our code is available at https://github.com/lnsmith54/BOSS to facilitate replication.

## 1. Introduction

In recent years, deep learning has achieved state-of-the-art performance for computer vision tasks such as image classification. However, a major barrier to the wide-spread adoption of deep neural networks for new applications is that training state-of-the-art deep networks typically requires thousands to millions of labeled samples to perform at high levels of accuracy and to generalize well.

Unfortunately, manual labeling is labor-intensive and might not be practical if labeling the data requires specialized expertise, such as in medical, defense, and scientific applications. In typical real-world scenarios for deep learning, one often has access to large amounts of unlabeled data but lacks the time or expertise to label the required massive numbers needed for training, validation, and testing. An ideal solution might be to achieve performance levels that are equivalent to fully supervised trained networks with only one manually labeled image per class.

In this paper, we investigate the potential for building one-shot semi-supervised (BOSS) learning up to achieve comparable performance as fully supervised training. To date, one-shot semi-supervised learning has been little studied and viewed as difficult. We build on the recent observation that one-shot semi-supervised learning is plagued by class imbalance problems (Smith and Conovaloff, 2020). In our context, class imbalance refers to a trained network with near 100% accuracy on a subset of classes and poor performance on other classes. We strongly advocate in classification tasks that practitioners evaluate and analyze test accuracies for every class, rather than only the average accuracy (Smith and Conovaloff, 2020; Fu et al., 2022). However, we are the first to apply data imbalance methods to unlabeled data.

Specifically, we demonstrate that good prototypes are crucial for successful semi-supervised learning and propose a prototype refinement method for the poorly performing classes. Also, we make use of the state-of-the-art in semi-supervised learning methods (i.e., FixMatch, Sohn et al., 2020) in our experiments. To combat class imbalance, we tested several variations of methods found in the literature for data imbalance problems (Johnson and Khoshgoftaar, 2019), which refers to the situation where the number of training samples per class varies substantially. We are the first to demonstrate that these methods significantly boost the performance of one-shot semi-supervised learning. Combining these methods with self-training (Rosenberg et al., 2005) makes it possible for CIFAR-10 and SVHN to attain comparable performance as fully supervised trained deep networks with 50 K and 73 K labeled training images, respectively.

Our contributions are:

1. We rigorously demonstrate for the first time the potential for one-shot semi-supervised learning to reach test accuracies with CIFAR-10 and SVHN that are comparable to fully supervised learning.
2. We propose the concept of class balancing on unlabeled data and investigate their value for one-shot semi-supervised learning. We introduce a novel measure of minority and majority classes and propose four class balancing methods that improve the performance of semi-supervised learning.
3. We investigate the causes of poor performance and hyper-parameter sensitivity. We hypothesize two causes and demonstrate solutions that improve performance.

## 2. Related Work

### 2.1. Semi-Supervised Learning

Semi-supervised learning is a hybrid between supervised and unsupervised learning, which combines the benefits of both and is better suited to real-world scenarios where unlabeled data is abundant. As with supervised learning, semi-supervised learning defines a task (i.e., classification) from labeled data but typically it requires much fewer labeled samples. In addition, semi-supervised learning leverages feature learning from unlabeled data to avoid overfitting the limited labeled samples. Semi-supervised learning is a large and mature field and there are several surveys and books on semi-supervised learning methods (Zhu, 2005; Chapelle et al., 2009; Zhu and Goldberg, 2009; Van Engelen and Hoos, 2020) for the interested reader. In this Section we mention only the most relevant of recent methods.

Recently there have been a series of papers on semi-supervised learning from Google Research, including MixMatch (Berthelot et al., 2019b), ReMixMatch (Berthelot et al., 2019a), and FixMatch (Sohn et al., 2020). MixMatch combines consistency regularization with data augmentation (Sajjadi et al., 2016), entropy minimization (i.e., sharpening) (Grandvalet and Bengio, 2005), and mixup (Zhang et al., 2017). ReMixMatch improved on MixMatch by incorporating distribution alignment and augmentation anchors. Augmentation anchors are similar to pseudo-labeling. FixMatch is the most recent and demonstrated state-of-the-art semi-supervised learning performance. In addition, the FixMatch paper has a discussion on one-shot semi-supervised learning with CIFAR-10.

The FixMatch algorithm (Sohn et al., 2020) is primarily a combination of consistency regularization (Sajjadi et al., 2016; Zhai et al., 2019) and pseudo-labeling (Lee, 2013). Consistency regularization utilizes unlabeled data by relying on the assumption that the model should output the same predictions when fed perturbed versions as on the original image. Consistency regularization has recently become a popular technique in unsupervised, self-supervised, and semi-supervised learning (Zhai et al., 2019; Van Engelen and Hoos, 2020). Several researchers have observed that strong data augmentation should not be used when inferring pseudo-labels for the unlabeled data but should be employed for consistency regularization (Xie et al., 2019; Sohn et al., 2020). Pseudo-labeling is based on the idea that one can use the model to obtain artificial labels for unlabeled data by retaining pseudo-labels for samples whose probability are above a predefined threshold.

A recent survey of semi-supervised learning (Van Engelen and Hoos, 2020) provides a taxonomy of classification algorithms. One of the methods in semi-supervised learning is self-training iterations (Rosenberg et al., 2005; Triguero et al., 2015) where a classifier is iteratively trained on labeled data plus high confidence pseudo labeled data from previous iterations. In our experiments we found that self-training provided a final boost to make the performance comparable to supervised training with the full labeled training dataset.

Unlike this paper, recent papers on semi-supervised learning, such as SimPLE Hu et al. (2021) and CoMatch Li et al. (2021), do not show results for one-shot semi-supervised learning. The SimPLE method uses at least 1,000 labels for CIFAR-10 and SVHN. On the other hand, CoMatch provides experiments on CIFAR-10 with as little as 20 labels but their reported performance is significantly lower than the performance obtained with the full labeled training dataset. There is one recent paper Lucas et al. (2021) that reports results for one-shot semi-supervised learning for CIFAR-10 and CIFAR-100. They too compare their results to FixMatch. Unlike our work, the performance they report is much lower than the fully-supervised performance.

### 2.2. Class Imbalance

Smith and Conovaloff (Smith and Conovaloff, 2020) demonstrated that in one-shot semi-supervised learning there are large variation in class performances, with some classes achieving near 100% test accuracies while other classes near 0% accuracies. That is, strong classes starve the weak classes, which is analogous to the class imbalance problem (Johnson and Khoshgoftaar, 2019). This observation suggests an opportunity to improve the overall performance by actively improving the performance of the weak classes.

We borrowed techniques from the literature on training with imbalanced data (Sun et al., 2007; Wang and Yao, 2012; Johnson and Khoshgoftaar, 2019) (i.e., some classes having many more training samples than other classes) to experiment with several methods for improving the performance of the weak classes with unlabeled data. However, with unlabeled data, labels to define the ground truth as to minority and majority classes do not exist. In this paper, we propose using the pseudo-labels as a surrogate to the ground truth for example class counting. Our experiments demonstrate that combining the counting of the pseudo-labels and methods for handling data imbalance substantially improves performance.

Methods for handling class imbalance can be grouped into two categories: data-level and algorithm-level methods. Data-level techniques (Wang and Yao, 2012) reduce the level of imbalance by undersampling the majority classes and oversampling the minority classes. Algorithm-level techniques (Sun et al., 2007) are commonly implemented with smaller loss factor weights for the training samples belonging to the majority classes and larger weights for the training samples belonging to the minority classes. In our experiments we tested variations of both types of methods and a hybrid of the two.

### 2.3. Meta-Learning

Our scenario superficially bears similarity to few-shot meta learning (Koch et al., 2015; Vinyals et al., 2016; Finn et al., 2017; Snell et al., 2017), which is a highly active area of research. The majority of the work in this area relies on a large labeled dataset with similar data statistics but this can be an onerous requirement for new applications. While there are some recent efforts in unsupervised pre-training for few-shot meta learning (Hsu et al., 2018; Antoniou and Storkey, 2019), our experiments with these methods demonstrated their inability to adequately perform in one-shot learning to bootstrap our process. Specifically, unsupervised one-shot learning with only five classes obtained a test accuracy of about 50% on high confidence samples and the accuracy dropped sharply when increasing the number of classes.

## 3. BOSS Methodology

### 3.1. FixMatch

Since we build on FixMatch (Sohn et al., 2020), we briefly describe the algorithm and adopt the formalism used in the original paper. For an N-class classification problem, let us define χ = {(*x*_*b*_, *y*_*b*_):*b*∈(1, …, *B*)} as a batch of B labeled examples, where *x*_*b*_ are the training examples and *y*_*b*_ are their labels. We also define $U=\{u_{b}:b\in (1,...,\mu )\}$ as a batch of μ unlabeled examples where μ = *r*_*u*_*B* and *r*_*u*_ is a hyperparameter that determines the ratio of U to χ. Let *p*_*m*_(*y*|*x*) be the predicted class distribution produced by the model for input *x*_*b*_. We denote the cross-entropy between two probability distributions *p* and *q* as *H*(*p, q*).

The loss function for FixMatch consists of two terms: a supervised loss *L*_*s*_ applied to labeled data and an unsupervised loss *L*_*u*_ for the unlabeled data. *L*_*s*_ is the cross-entropy loss on weakly augmented labeled examples:


(1) $$\begin{array}{r} L_{s}=\frac{1}{B}\overset{B}{\underset{b=1}{\sum }}H\left(y_{b},p_{m}\left(y|\alpha \left(x_{b}\right)\right)\right) \end{array}$$

where α(*x*_*b*_) represents weak data augmentation on labeled sample *x*_*b*_.

For the unsupervised loss, the algorithm computes the label based on weakly augmented versions of the image as *q*_*b*_ = *p*_*m*_[*y*|α(*u*_*b*_)]. It is essential that the label is computed on weakly augmented versions of the unlabeled training samples and not on strongly augmented versions. The pseudo-label is computed as $\hat{q_{b}}=\arg\max\left(q_{b}\right)$ and the unlabeled loss is given as:


(2) $$\begin{array}{r} L_{u}=\frac{1}{\mu }\overset{\mu }{\underset{b=1}{\sum }}1\left(max\left(q_{b}\right)\geq \tau \right)H\left(\hat{q_{b}},p_{m}\left(y|A\left(u_{b}\right)\right)\right) \end{array}$$

where $A\left(u_{b}\right)$ represents applying strong augmentation to sample *u*_*b*_ and τ is a scalar confidence threshold that is used to include only high confidence terms. The total loss is given by *L* = *L*_*s*_+λ_*u*_*L*_*u*_ where λ_*u*_ is a scalar hyper-parameter. Additional details on the FixMatch algorithm are available in the original paper (Sohn et al., 2020).

### 3.2. Prototype Refining

Previous work by Sohn et al. on one-shot semi-supervised learning relied on the dataset labels to randomly choose an example for each class. The authors demonstrated that the choice of these samples significantly affected the performance of their algorithm. Specifically, they ordered the CIFAR-10 training data by how representative they were of their class by utilizing fully supervised trained models and found that using more prototypical examples achieved a median accuracy of 78% while the use of poorly representative samples failed to converge at all. The authors acknowledged that their method for finding prototypes was not practical. In contrast, we now present a practical approach for choosing an iconic prototype for each class.

In real-world scenarios, one's data is initially all unlabeled but it is not overly burdensome for an expert to manually sift through some of their dataset to find one iconic example of each class. In choosing iconic images of each class, the labeler's goal is to pick images that represent the class objects well, while minimizing the amount of background distractors in the image. While the labeler is choosing the most iconic examples to be class prototypes for one-shot training of the network, it is beneficial to designate the less representative examples as part of a validation or test dataset. In our own experiments with labeled datasets CIFAR-10 and SVHN, we did not rely on the training labels but reviewed a small fraction of the training data to manually choose class prototypes.

In addition, we also propose a simple iterative technique for improving the choice of prototypes because good prototypes are important to good performance. After choosing prototypes, the next step is to make a training run and examine the class accuracies. For any class with poor accuracy relative to the other classes, it is likely that a better prototype can be chosen. We recommend returning to the unlabeled or test datasets to find replacement prototypes for only the poorly performing classes. In our experiments we found doing this even once to be beneficial.

One might argue that prototype refining is as much work as labeling several examples per class and using many training samples will make it easier to train the model. From only a practical perspective, labeling 5 or 10 examples per class is not substantially more effort relative to labeling only one iconic example per class and prototype refining. While in practice one may want to start with more than one example for ease of training, there are scientific, educational, and algorithmic benefits to studying one-shot semi-supervised learning, which we discuss in our Appendix. Also, non-representative examples can be included in a labeled test or validation dataset for use in evaluating the quality of the training.

### 3.3. Class Balancing

We believe a class imbalance problem is an important factor in training neural networks, not only in one-shot semi-supervised learning but also a factor for small to mid-sized datasets. It is typical that a network with random weights usually outputs a single class label for every sample (i.e., randomly initialized networks do not generate random predictions). Hence, all networks start their training with elements of the class imbalance problem but the presence of large, balanced training data allows the network to overcome this problem. Since class imbalance is always present when training deep networks, class balancing methods might always be valuable, particularly when training on one-shot, few-shot, or small labeled datasets, and we leave further investigations of this for future work.

Unlike the data imbalance domain, the ground truth imbalance proportions are unknown with unlabeled datasets. Our innovation here is to use the model generated pseudo-labels as a surrogate for class counting and estimating class imbalance ratios (i.e., determining majority and minority classes). Specifically, as the algorithm computes the pseudo-labels for all of the unlabeled training samples, it counts the number that fall within each class, which we designate as $C=\left\{c_{n}:n\in \left(1,...,N\right)\right\}$ where *N* is the number of classes. We assume a similar number of unlabeled samples in each class so the number of pseudo-labels in each class should also be similar.

Our first class balancing method is based on oversampling minority classes. Our algorithm reduces the pseudo-labeling thresholds for minority classes to include more examples of the minority classes in the training. Formally, in pseudo-labeling the following unsupervised loss function is used for the unlabeled data in place of Equation (2):


(3) $$\begin{array}{r} L_{u}=\frac{1}{\mu }\overset{\mu }{\underset{b=1}{\sum }}1\left(max\left(q_{b}\right)\geq \tau_{n}\right)H\left(\hat{q_{b}},q_{b}\right) \end{array}$$

where $q_{b}=p_{m}\left[y|A\left(u_{b}\right)\right]$, $\hat{q_{b}}=\arg\max\left(q_{b}\right)$, and τ_*n*_ is the class dependent threshold for inclusion in the unlabeled loss *L*_*u*_. We define the class dependent thresholds as:


(4) $$\begin{array}{r} \tau_{n}=\tau -\Delta \left(1-\frac{c_{n}}{max\left(C\right)}\right) \end{array}$$

where *c*_*n*_ is the number of pseudo-labeled in class n,max(C) is the maximum count of all the classes, and Δ is a scalar hyper-parameter (τ>Δ>0) guiding how much to lower the threshold for minority classes. Hence, the most frequent class will use a threshold of τ while minority classes will use lower thresholds, down to τ−Δ.

The next two class balancing methods are variations on loss function class weightings. In the FixMatch algorithm, all unlabeled samples above the threshold are included in Equation (3) with the same weight. Instead, our second class balancing algorithm becomes:


(5) $$\begin{array}{r} L_{u}=\frac{1}{Z\mu }\overset{\mu }{\underset{b=1}{\sum }}1\left(max\left(q_{b}\geq \tau_{n}\right)\right)H\left(\hat{q_{b}},q_{b}\right)/c_{n} \end{array}$$

where the loss terms are divided by *c*_*n*_ and *Z* is a normalizing factor that makes *L*_*u*_ the same magnitude as without this weighting scheme (this allows the unlabeled loss weighting λ_*u*_ to remain the same).

Our third class balancing algorithm is identical to the previous method except it uses an alternate class count ĉ_*u*_ in Equation (5). Here we define ĉ_*u*_ using only the high confidence pseudo-labeled samples (i.e., samples that are above the threshold). The intuition of this third method is that each of the classes should contribute equally to the loss *L*_*u*_ (i.e., each sample's loss is divided by the number of samples of that class included in *L*_*u*_). In practice, this method's weights might be an order of magnitude larger than the previous method's weights, which might contribute to training instability, so we compare both methods in Section 4.2.

Our fourth class balancing algorithm is a hybrid of the data and algorithmic methods. Specifically, it is a combination of our class balancing methods 1 and 3. Our experiments with this hybrid method demonstrates the benefits of combining the class balancing methods.

### 3.4. Self-Training Iterations

Labeled and unlabeled data play different roles in semi-supervised learning. Here we propose self-training iterations where the pseudo-labels of the highest confidence unlabeled training samples are combined with labeled samples in a new iteration. Increasing the number of labeled samples per class improves performance, and substantially reduces training instability and performance variability. Although some of these pseudo-labels might be wrong, we rely on the observation that the training of deep networks are robust to small amounts of labeling noise. Hence, we aimed to achieve a 90% accuracy from the first iteration of semi-supervised learning with the class balancing methods.

Self-training in BOSS adds to the testing stage a computation of the model predictions on all of the unlabeled training data. These are sorted from the highest prediction probabilities down and the dataset is saved. After the original training run, the labeled data can be combined with a number of the highest prediction samples from each class and a subsequent self-training iteration run can use the larger labeled dataset for retraining a new network. We experimented with labeling 5, 10, 20, and 40 of the top predictions per class and the results are reported in Section 4.3.

## 4. Experiments

In this section, we demonstrate that the BOSS algorithms can achieve comparable performance with fully-supervised training of CIFAR-10^1^ (Krizhevsky and Hinton, 2009) and SVHN^2^ (Netzer et al., 2011). We compare our results to FixMatch^3^ (Sohn et al., 2020) and demonstrate the value of our approach. Our experiments use a Wide ResNet-28-2 (Zagoruyko and Komodakis, 2016) that matches the FixMatch reported results and we used the same cosine learning rate schedule described by Sohn et al. (2020). We repeated our experiments with a ShakeNet model (Gastaldi, 2017) and obtained similar result that lead to the same insights and conclusions. Our hyper-parameters were in a small range and the specifics are provided in the Appendix. For data and data augmentation, we used the default augmentation in FixMatch but additional experiments (not shown) did show that using RandAugment (Cubuk et al., 2019) for strong data augmentation provides a slight improvement. Our runs with fully supervised learning of the Wide ResNet-28-2 model produced a test accuracy of 94.9±0.3% for CIFAR-10 (Krizhevsky and Hinton, 2009) and test accuracy of 98.26±0.04% for SVHN (Netzer et al., 2011), which we use for our basis of comparison. Our code is available at https://github.com/lnsmith54/BOSS to facilitate replication and for use with future real-world applications.

### 4.1. Choosing Prototypes and Prototype Refining

For our experiments with CIFAR-10, we manually reviewed the first few hundred images and choose five sets of prototypes that we will refer to as class prototype sets 1–5. However, the practioner need only create one set of class prototypes and can perform prototype refining, as we describe below.

Table 1 presents the averaged (over two runs) test accuracies for each class, computed from FixMatch on the CIFAR-10 test dataset for each of the prototype sets 1–5. This table illustrates that a good choice of prototypes (i.e., set = 3) can lead to good performance in most of the classes, which enables a good overall performance. Table 1 also shows that for other sets the class accuracies can be quite high for some classes while low for other classes. Hence, the poor performance of some classes implies that the choice of prototypes for these classes in those sets can be improved. In prototype refining, one simply reviews the class accuracies to find which prototypes should be replaced.

**Table 1:** Class accuracies.

**Set**	**Airplane**	**Auto**	**Bird**	**Cat**	**Deer**	**Dog**	**Frog**	**Horse**	**Ship**	**Truck**	**Mean**
1	29	98	71	89	97	16	98	97	97	97	79
2	28	99	70	43	97	89	98	97	98	0	72
3	96	98	63	20	97	96	98	87	98	97	86
4	29	98	65	10	96	32	98	97	97	96	72
5	28	97	70	46	96	48	53	76	96	97	72
6	80	98	71	52	97	92	98	87	98	97	82
7	28	99	75	54	95	86	95	86	96	94	83

*One-shot semi-supervised average (of 2 runs) class accuracies for CIFAR-10 test data with the FixMatch model, that was trained on sets of manually chosen prototypes for each class. Prototype set 6 was modified from set 2 and prototype set 7 was modified from set 4 (i.e., prototype refining)*.

We demonstrate prototype refining with two examples. The airplane and truck class accuracies in set 2 are poor so we replaced these two prototypes and name this set 6. In set 4, the cat and dog classes are performing poorly so we replaced these two prototypes and name this set 7. Table 1 shows the class accuracies for sets 6 and 7 and these results are better than the original sets; that is, prototype refining of these two sets raised the overall test accuracies from 72 up to 82–83%.

### 4.2. Class Balancing

In this section, we report the results from FixMatch and demonstrate substantial improvements with the class balancing methods in BOSS. Table 2 presents our main results for CIFAR-10, which illustrates the benefits from prototype refining, class balancing, and one self-training iteration. The first five rows in the table list the results for the five sets of class prototypes (i.e., 1 prototype per class) for CIFAR-10. Rows for sets 6 and 7 provide the results for prototype refining of the original sets 2 and 4, respectively. The FixMatch column shows results (i.e., average and standard deviation over four runs) for the original FixMatch code on the prototype sets.

**Table 2:** Main results.

		**BOSS balance method**				**Self-training**			
**Set**	**FixMatch**	**1**	**2**	**3**	**4**	**+5**	**+10**	**+20**	**+40**
1	79 ± 1	**91.4 ± 2**	90 ± 5	84 ± 6	88 ± 2	94.8	95.2	95.2	95.2
						±0.1	±0.1	±0.1	±0.1
2	74 ± 5	**91.8 ± 1**	90 ± 3	88 ± 2	80 ± 14	93.6	95.1	95.1	95.1
						±0.2	±0.1	±0.3	±0.2
3	86 ± 1	92.8 ± 0.2	91 ± 2	91 ± 3	**92.8 ± 0.1**	94.6	94.8	94.9	95.2
						±0.5	±0.5	±0.1	±0.1
4	74 ± 8	77.7 ± 0.3	81 ± 6	81 ± 8	**90 ± 7**	94.9	94.9	94.9	95.1
						±0.1	±0.4	±0.5	±0.3
5	69 ± 7	86 ± 7	89 ± 6	83 ± 10	**90 ± 3**	89.6	95.2	95.2	95.2
						±0.3	±0.1	±0.2	±0.1
6	82 ± 0.6	91.5 ± 1	92 ± 0.7	91.8 ± 1	**92 ± 1**	94.6	95.1	94.7	94.9
						±0.1	±0.2	±0.1	±0.1
7	78 ± 0.1	91.7 ± 0.3	92.3 ± 0.8	91.1 ± 2.5	**93 ± 0.3**	94.9	94.7	94.9	95.1
						±0.1	±0.2	±0.1	±0.1

*BOSS methods are compared using five sets of class prototypes (i.e., 1 prototype per class) for CIFAR-10, plus two sets from prototype refining. The FixMatch column shows test accuracies (average and standard deviation of 4 runs) for the original FixMatch code on the prototype sets. The next four columns give the accuracy results for the class balance methods (see text for a description of class balance methods). Results for the PyTorch reimplementation of FixMatch and modified with the BOSS methods are shown in brackets [.]. The self-training iteration was performed with the top pseudo-labels from the run shown in bold and the results are in the next four columns*.

The next four columns present the BOSS results with class balancing methods. As described in Section 3.3, class balance method 1 represents oversampling of minority classes, balance methods 2 and 3 are two forms of class-based loss weightings, and balance = 4 is a hybrid that combines balance methods 1 and 3. The use of class balancing significantly improves on the original FixMatch results, with increases of up to 20 absolute percentage points. Generally, the hybrid class balance method 4 is best, except when instabilities hurt the performance. The performance is generally in the 90% range with good performance across all the classes, which enables the self-training iteration to bump the accuracies to be comparable to the test accuracy from supervised training on the full labeled training dataset.

Table 2 indicates that good class prototypes (i.e., sets 3, 6, and 7) result in test accuracies near 90% and low variance between runs. However, when some of the class prototypes are inferior, some of the training runs exhibit instabilities that cause lower averaged accuracies and higher variance. We provide a discussion in Section 4.5 on the cause of these instabilities and on how to improve these results.

### 4.3. Self-Training Iterations

The final four columns of Table 2 list the results of performing one self-training iteration. The self-training was initialized with the original single labeled sample per class, plus the most confident pseudo-labeled examples from the BOSS training run that is highlighted in bold. For example, the “+5” columns means that five pseudo-labeled examples per class were combined with the original labeled prototypes to make a set with a total of 60 labeled examples. These self-training results demonstrate that one-shot semi-supervised learning can reach comparable performance to the results from fully supervised training (i.e., 94.9%), often with adding as few as five samples per class. However, we expect that in practice, self-training by adding more samples per class will prove more reliable.

### 4.4. SVHN

SVHN is obtained from house numbers in Google Street View images and is used for recognizing digits (i.e., 0–9) in natural scene images. Visual review of the images show that the training samples are of poor quality (i.e., blurry) and often contain distractors (i.e., multiple digits in an image). Because of the quality issue, we needed to review several hundred unlabeled training samples in order to find four class prototype sets that are reported in Table 3. Even though the SVHN training images are of poorer quality than the CIFAR-10 training images, one-shot semi-supervised learning with FixMatch on sets of prototypes produced higher test accuracies than with CIFAR-10. Table 3 presents equivalent results for the SVHN dataset as those results that were reported in Table 2 for CIFAR-10. Since the results for FixMatch are all above 89%, we did not perform prototype refining on any of these sets. However, here too the class balancing methods increase the test accuracies above the FixMatch results. With these four class prototype sets, class balance method 1 produces the best results. The test accuracies from balance method 1 are ~1% lower than the fully supervised results of 98.26±0.04%. The improvements from self-training were small and the best results fell about 0.5% below the results of fully supervised training. We believe the differences between CIFAR-10 and SVHN are related to the natures of the datasets.

**Table 3:** SVHN.

		**BOSS balance method**				**Self-training**			
**Set**	**FixMatch**	**1**	**2**	**3**	**4**	**+5**	**+10**	**+20**	**+40**
1	95.9 ± 3	**97.4 ± 0.2**	96.4 ± 0.9	95.7 ± 1.6	96.8 ± 0.1	97.9	97.9	97.9	97.8
2	91.5 ± 3	**97.4 ± 0.1**	97.1 ± 0.1	97.1 ± 0.1	95.6 ± 0.1	94.1	97.9	97.6	97.7
3	93.9 ± 0.1	**97.3 ± 0.3**	97.2 ± 0.2	92 ± 7	91.3 ± 0.3	97.8	97.9	97.8	97.9
4	89.2 ± 12	**96.5 ± 0.6**	90 ± 10	89 ± 11	83 ± 16	97.6	96.7	97.0	98.0

*BOSS methods are compared using four sets of class prototypes (i.e., 1 prototype per class) for SVHN. The FixMatch column shows results for the original FixMatch code on the prototype sets. The next four columns give the accuracy results for the class balance methods. Results are an average of test accuracies for four runs. The self-training iteration was performed on the results from the class balancing shown in bold*.

### 4.5. Investigation of Training Instabilities

In our experiments we observed high sensitivity of one-shot semi-supervised learning performance to the choices for the hyper-parameters and the class prototype sets, which motivated us to investigate this matter in greater depth. That is, we observed that good choices for the prototypes and prototype refining significantly reduced the instabilities and the variability of the results (i.e., few instabilities were encountered for CIFAR-10 prototype sets 3, 6, and 7 so the final accuracies were higher and the standard deviations of the results were lower). In sets where the performance was inferior, there was always at least one class that performed poorly. In addition, we found a high sensitivity to the hyper-parameter values, which made a significant difference in the results.

**Figure 1:**
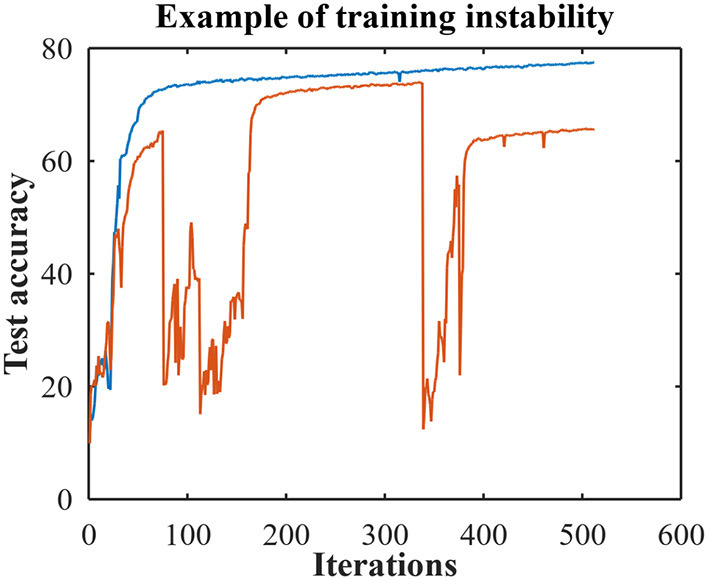
An example of training to a poor local minimum (blue) and training with instabilities (red). Both end with poor test accuracies but for different reasons.

We investigated the cases of poor performance and discovered that there were two different situations. Figure 1 provides examples of test accuracies during the training for both situations. The blue curve is the test accuracy where in one training run the network learns a final test accuracy of 77%. We hypothesize that in this situation the network can get stuck in a poor local minimum that is due to poor prototype choices and can be improved with prototype refining or by hyper-parameter fine tuning. The red curve in Figure 1 is an example of the other case and here the training is dominated by instabilities (i.e., where the model suddenly diverges during training) and the final test accuracy is 65%. Interestingly, we found that it is important when tuning the hyper-parameters to identify which scenario is occurring.

Our experiments with training instabilities (i.e., the red curve) implied that they can be caused by too much class balancing. We hypothesize that when the model struggles to classify some of the classes, the class balancing methods can force the pseudo-labeling to mislabel samples in order to have the appearance of class balance. In these cases, it is better to reduce the amount of class balancing by using a smaller value for Δ for class balance methods 1 and 4, and using a smaller value for λ_*u*_ for class balance methods 2 and 3. In addition, we observed that decreasing weight decay (WD) and the learning rate (LR) improves performance when there are instabilities.

On the other hand, if the inferior performance is due to poor local minimum (i.e., the blue curve), one can either improve the class prototypes (i.e., prototype refining) or increase the amount of class balancing. This is the opposite of what should do for instabilities; that is, one can use a larger value for Δ for class balance methods 1 and 4, use a larger value for λ_*u*_ for class balance methods 2 and 3, or increase weight decay (WD) and the learning rate (LR). We also observed that it helps to increase τ if there are instabilities and to decrease τ in the poor local minimum situation.

Table 4 demonstrates how to improve the results presented in Table 2 (for consistency we used the same hyper-parameter values for all of the class balance runs shown in Table 2). Table 4 contains results of hyper-parameter fine tuning where we reported earlier test accuracies below 85%. We list the class prototype set (Set), the BOSS class balancing method (Balance), weight decay (WD), initial learning rate (LR), the change in the confidence threshold for minority classes (Δ), the unlabeled loss multiplicative factor (λ_*u*_), the confidence threshold (τ), and the final test accuracy. Furthermore, we provide a short description that indicates if the training curve displays instabilities (i.e., the red curve in Figure 1) or a poor local minimum (i.e., the blue curve). Or the description points out the hyper-parameters that were tuned to improve the performance.

**Table 4:** Illustration of the sensitivity to the hyper-parameters WD, LR, Δ, λ_*u*_, and τ. See the text for guidance on how to tune these hyper-parameters for situations with inferior performance due to instabilities or local minimums.

**Set**	**Balance**	**Description**	**WD**	**LR**	**Δ**	**λ_*u*_**	**τ**	**Accuracy (%)**
1	3	Instabilities	8 × 10^−4^	0.06	0	1	0.9	84 ± 6
1	3	Decrease λ_*u*_	8 × 10^−4^	0.06	0	0.5	0.9	87 ± 1
2	4	Instabilities	8 × 10^−4^	0.06	0.25	1	0.95	80 ± 14
2	4	Decrease Δ, WD, LR	6 × 10^−4^	0.04	0.1	1	0.95	94.5 ± 0.1
4	1	Local min	8 × 10^−4^	0.06	0.25	1	0.9	77.5 ± 0.1
4	1	Increase Δ, τ	8 × 10^−4^	0.06	0.3	1	0.95	93.2 ± 0.2
4	2	Local min	8 × 10^−4^	0.06	0	1	0.9	81 ± 6
4	2	Increase λ_*u*_	8 × 10^−4^	0.06	0	2	0.9	92 ± 2
4	3	Local min	8 × 10^−4^	0.06	0	1	0.9	81 ± 8
4	3	Increase λ_*u*_	8 × 10^−4^	0.06	0	2	0.9	88 ± 3
5	1	Instabilities	8 × 10^−4^	0.06	0.25	1	0.95	86 ± 7
5	1	Decrease Δ	8 × 10^−4^	0.06	0.1	1	0.95	90.7 ± 0.1
5	2	Instabilities	8 × 10^−4^	0.06	0	1	0.9	89 ± 6
5	2	Decrease λ_*u*_	8 × 10^−4^	0.06	0	0.75	0.9	91.7 ± 1
5	3	Instabilities	8 × 10^−4^	0.06	0	1	0.9	83 ± 10
5	3	Decrease WD, LR	6 × 10^−4^	0.04	0	1	0.9	93.5 ± 2

The examples in Table 4 show improved results for both the problem of instability and for poor local minimums. The examples include modifying Δ, weight decay, learning rate, and τ. In most cases the final accuracies are improved substantially with small changes in the hyper-parameter values. This demonstrates the sensitivity of one-shot semi-supervised learning to hyper-parameter values.

While this sensitivity can be challenging in practice, we note that this sensitivity can also lead to new opportunities. For example, often researchers propose new network architectures, loss functions, and optimization functions that are tested in the fully supervised regime where small performance gains are used to claim a new state-of-the-art. If these algorithms were instead tested in one-shot semi-supervised learning, more substantial differences in performance would better differentiate methods. Along these lines, we also advocate the use of one-shot semi-supervised learning with AutoML and neural architecture search (NAS) (Elsken et al., 2018) to find optimal hyper-parameters and architectures.

## 5. Conclusions

The BOSS methodology relies on simple concepts: choosing iconic training samples with minimal background distractors, employing class balancing techniques, and self-training with the highest confidence pseudo-labeled samples. Our experiments in Section 4 demonstrate the potential of training a network with only one sample per class and we have confirmed the importance of class balancing methods. While our methods have limitations (as discussed in the Appendix), this paper breaks new ground in one-shot semi-supervised learning and attains high performance. BOSS brings one-shot and few-shot semi-supervised learning closer to reality.

We proposed the novel concept of class balancing on unlabeled data. We introduced a novel way to measure class imbalance with unlabeled data and proposed four class balancing methods that improve the performance of semi-supervised learning. In addition, we investigated hyper-parameter sensitivity and the causes for weak performance (i.e., training instabilities), where we proposed two opposite sets of solutions.

Our work provides researchers with the following observations and insights:

1. There is evidence that labeling a large number of samples might not be necessary for training deep neural networks to high levels of performance.
2. All networks have a class imbalance problem to some degree. Examining class accuracies relative to each other provides insights into the network's training.
3. Each training sample can affect the training. One-shot semi-supervised learning provides a mechanism to study the atomic impact of a single sample. This opens up the opportunity to investigate the factors in a sample that help or hurt training performance.

Training neural networks for image classification with only one labeled example per class remain a barely studied field. Future work includes applying the BOSS methodology to more complex image classification datasets, such as ImageNet and STL-10, which has not been investigated as far as we know. While we do not expect to reach the same test accuracies as with the fully supervised training, we do anticipate substantial gains can be possible. Our work lays the foundation for one-shot learning and opens the door to future research.

## Data Availability Statement

The original contributions presented in the study are included in the article/Supplementary Material, further inquiries can be directed to the corresponding author/s.

## Author Contributions

AC tested the final software to verify the reproducibility of the results in this article and reviewed the content of this manuscript. Everything else related to this investigation and manuscript was done by LS. All authors contributed to the article and approved the submitted version.

## Conflict of Interest

The authors declare that the research was conducted in the absence of any commercial or financial relationships that could be construed as a potential conflict of interest.

## Publisher's Note

All claims expressed in this article are solely those of the authors and do not necessarily represent those of their affiliated organizations, or those of the publisher, the editors and the reviewers. Any product that may be evaluated in this article, or claim that may be made by its manufacturer, is not guaranteed or endorsed by the publisher.

## Footnotes

^1^Available at https://www.cs.toronto.edu/~kriz/cifar.html.

^2^Available at http://ufldl.stanford.edu/housenumbers/.

^3^With appreciation, we acknowledge the use of the code kindly provided by the authors at https://github.com/google-research/fixmatch.

## References

[B1] Antoniou A. Storkey A. (2019). Assume, augment and learn: unsupervised few-shot meta-learning via random labels and data augmentation. arXiv [Preprint]. arXiv:1902.09884. 10.48550/arXiv.1902.09884

[B2] Berthelot D. Carlini N. Cubuk E. D. Kurakin A. Sohn K. Zhang H.. (2019a). Remixmatch: semi-supervised learning with distribution alignment and augmentation anchoring. arXiv [Preprint]. arXiv:1911.09785. 10.48550/arXiv.1911.09785

[B3] Berthelot D. Carlini N. Goodfellow I. Papernot N. Oliver A. Raffel C. A. (2019b). “Mixmatch: a holistic approach to semi-supervised learning,” in Advances in Neural Information Processing Systems, Vancouver, 5050–5060.

[B4] Chapelle O. Scholkopf B. Zien A. (2009). Semi-supervised learning (chapelle, o. et al., eds.; 2006)[book reviews]. IEEE Trans. Neural Netw. 20, 542–542. 10.1109/TNN.2009.2015974

[B5] Cubuk E. D. Zoph B. Shlens J. Le Q. V. (2019). Randaugment: practical data augmentation with no separate search. arXiv preprint arXiv:1909.13719. 10.1109/CVPRW50498.2020.00359

[B6] Elsken T. Metzen J. H. Hutter F. (2018). Neural architecture search: a survey. arXiv preprint arXiv:1808.05377. 10.1007/978-3-030-05318-5_3

[B7] Finn C. Abbeel P. Levine S. (2017). “Model-agnostic meta-learning for fast adaptation of deep networks,” in Proceedings of the 34th International Conference on Machine Learning, Sydney Australia, Vol. 70, 1126–1135.

[B8] Fu M. Cao Y.-H. Wu J. (2022). Worst case matters for few-shot recognition. arXiv [Preprint]. arXiv:2203.06574. 10.48550/arXiv.2203.06574

[B9] Gastaldi X. (2017). Shake-shake regularization. arXiv [Preprint]. arXiv:1705.07485. 10.48550/arXiv.1705.07485

[B10] Grandvalet Y. Bengio Y. (2005). “Semi-supervised learning by entropy minimization,” in Advances in Neural Information Processing Systems, Vancouver, 529–536.

[B11] Hsu K. Levine S. Finn C. (2018). Unsupervised learning via meta-learning. arXiv [Preprint]. arXiv:1810.02334. 10.48550/arXiv.1810.02334

[B12] Hu Z. Yang Z. Hu X. Nevatia R. (2021). “Simple: similar pseudo label exploitation for semi-supervised classification,” in Proceedings of the IEEE/CVF Conference on Computer Vision and Pattern Recognition, Nashvile, TN, 15099–15108. 10.1109/CVPR46437.2021.01485

[B13] Johnson J. M. Khoshgoftaar T. M. (2019). Survey on deep learning with class imbalance. J. Big Data 6:27. 10.1186/s40537-019-0192-5

[B14] Koch G. Zemel R. Salakhutdinov R. (2015). “Siamese neural networks for one-shot image recognition,” in ICML Deep Learning Workshop, Vol. 2 (Lille).

[B15] Krizhevsky A. Hinton G. (2009). Learning multiple layers of features from tiny images (Technical Report). University of Toronto.

[B16] Lee D.-H. (2013). “Pseudo-label: the simple and efficient semi-supervised learning method for deep neural networks,” in Workshop on Challenges in Representation Learning, ICML, Atlanta, GA, Vol. 3, 2.

[B17] Li J. Xiong C. Hoi S. C. (2021). “Comatch: semi-supervised learning with contrastive graph regularization,” in Proceedings of the IEEE/CVF International Conference on Computer Vision, Montreal, CA, 9475–9484. 10.1109/ICCV48922.2021.00934

[B18] Lucas T. Weinzaepfel P. Rogez G. (2021). Barely-supervised learning: semi-supervised learning with very few labeled images. arXiv preprint arXiv:2112.12004.

[B19] Netzer Y. Wang T. Coates A. Bissacco A. Wu B. Ng A. Y. (2011). “Reading digits in natural images with unsupervised feature learning,” in NIPS Workshop on Deep Learning and Unsupervised Feature Learning, vol. 2011 (Granada).

[B20] Rosenberg C. Hebert M. Schneiderman H. (2005). Semi-supervised self-training of object detection models. WACV/Motion 2. 10.1109/ACVMOT.2005.107

[B21] Sajjadi M. Javanmardi M. Tasdizen T. (2016). “Regularization with stochastic transformations and perturbations for deep semi-supervised learning,” in Advances in Neural Information Processing Systems, Barcelona, 1163–1171.

[B22] Smith L. N. Conovaloff A. (2020). Empirical perspectives on one-shot semi-supervised learning. arXiv [Preprint]. arXiv:2004.04141. 10.48550/arXiv.2004.04141

[B23] Snell J. Swersky K. Zemel R. (2017). “Prototypical networks for few-shot learning,” in Advances in Neural Information Processing Systems, Los Angeles, CA, 4077–4087.34495842

[B24] Sohn K. Berthelot D. Carlini N. Zhang Z. Zhang H. Raffel C. A.. (2020). Fixmatch: simplifying semi-supervised learning with consistency and confidence. Adv. Neural Inform. Process. Syst. 33, 596–608. 10.48550/arXiv.2001.07685

[B25] Sun Y. Kamel M. S. Wong A. K. Wang Y. (2007). Cost-sensitive boosting for classification of imbalanced data. Pattern Recogn. 40, 3358–3378. 10.1016/j.patcog.2007.04.009

[B26] Triguero I. García S. Herrera F. (2015). Self-labeled techniques for semi-supervised learning: taxonomy, software and empirical study. Knowl. Inform. Syst. 42, 245–284. 10.1007/s10115-013-0706-y

[B27] Van Engelen J. E. Hoos H. H. (2020). A survey on semi-supervised learning. Mach. Learn. 109, 373–440. 10.1007/s10994-019-05855-6

[B28] Vinyals O. Blundell C. Lillicrap T. kavukcuoglu K. Wierstra D. (2016). “Matching networks for one shot learning,” in Advances in Neural Information Processing Systems, Barcelona, 3630–3638.

[B29] Wang S. Yao X. (2012). Multiclass imbalance problems: analysis and potential solutions. IEEE Trans. Syst. Man Cybern. Part B 42, 1119–1130. 10.1109/TSMCB.2012.2187280 22438514

[B30] Xie Q. Hovy E. Luong M.-T. Le Q. V. (2019). Self-training with noisy student improves imagenet classification. arXiv preprint arXiv:1911.04252. 10.1109/CVPR42600.2020.01070

[B31] Zagoruyko S. Komodakis N. (2016). Wide residual networks. arXiv preprint arXiv:1605.07146. 10.5244/C.30.87

[B32] Zhai X. Oliver A. Kolesnikov A. Beyer L. (2019). “S4l: self-supervised semi-supervised learning,” in Proceedings of the IEEE International Conference on Computer Vision, Venice, 1476–1485. 10.1109/ICCV.2019.00156

[B33] Zhang H. Cisse M. Dauphin Y. N. Lopez-Paz D. (2017). mixup: beyond empirical risk minimization. arXiv [Preprint]. arXiv:1710.09412. 10.48550/arXiv.1710.09412

[B34] Zhu X. Goldberg A. B. (2009). Introduction to semi-supervised learning. Synthes. Lect. Artif. Intell. Mach. Learn. 3, 1–130. 10.2200/S00196ED1V01Y200906AIM006

[B35] Zhu X. J. (2005). Semi-Supervised Learning Literature Survey. Technical report, University of Wisconsin-Madison Department of Computer Sciences.

