# Supplementary material for: Building One-Shot Semi-Supervised (BOSS) Learning Up to Fully Supervised Performance
Source: Front Artif Intell. 2022 Jun 2;5:880729. doi: 10.3389/frai.2022.880729 (PMC9200967; doi:10.3389/frai.2022.880729)
Supplement: Supplementary file 1 [file Data_Sheet_1.pdf]

Appendix

1 BROADER IMPACT

It is widely accepted that large labeled datasets are an essential component of training deep neural networks, either directly for training or indirectly via transfer learning. To the best of our knowledge, this paper is the first to demonstrate performance comparable to fully supervised learning with one-shot semi-supervised learning. Eliminating the burden of labeling massive amounts of training data creates great potential for new neural network applications that attain high performance, which is especially important when labeling requires expertise. Hence, the societal impact will be to make deep learning applications even more widespread.

From a scientific perspective, one-shot semi-supervised learning provides important insights on the intricacies of training deep neural networks. The effect of changing just one training image can significantly impact the final performance. Unlike fully supervised learning which commonly deals with the training of large datasets, this method provides a technique to gain information about the impact of a single labeled sample in training. In addition, we anticipate that further investigation into the instability issues of one-shot semi-supervised learning will lead to new understandings of training neural networks.

Furthermore, the experience of training highly sensitive networks provides an educational experience on hyper-parameter tuning that carries over to easier training situations. In order to achieve convergence with one-shot semi-supervised learning, one must learn how to tune the hyper-parameters and architecture well. Similarly, we believe that utilizing one-shot semi-supervised learning with automatic methods such as AutoML and neural architecture search (NAS) will lead to better choices for hyper-parameters and architectures.

Limitations: While our work has taken valuable steps towards making one or few-shot semi-supervised learning possible for applications, a large gap still remains before this can be realized in practice, especially due to issues with stability during training and hyper-parameter sensitivity. The sensitivity of the results to choices of the hyper-parameters makes one-shot semi-supervised learning difficult to use in real-world applications. While there is a wide range of valuable applications (e.g., medical) that could benefit from semi-supervised learning, the testing of these applications is beyond the scope of this work.

While we attempted to provide a thorough investigation, there are a number of limitations in our work and several factors that we did not have sufficient time to explore. Our implementation was built on state-of-the-art FixMatch algorithm but the ideas presented here should carry over to other semi-supervised learning methods, such as the π model, temporal ensembling Laine and Aila (2017), VAT Miyato et al. (2018), ICT Verma et al. (2019), UDA Xie et al. (2019), S^4L Zhai et al. (2019), MixMatch Berthelot et al. (2019), and Mean Teachers Tarvainen and Valpola (2017) but this was not tested because none of these other methods have demonstrated results with less than 25 labeled examples per class. The model used in our experiments was a Wide ResNet-28-2 and the experiments were replicated with ShakeNet Gastaldi (2017) with analogous results, indicating that our conclusions and insights are independent of the model architecture.

In addition, we made use of labeled test data to demonstrate the performance of BOSS. In practical settings, one has a large unlabeled dataset and one wishes to avoid burdensome manual labeling. However, the samples in the test dataset are less important than the choices for the class prototypes, so a small test

Method	balance	weight decay	LR	Batch	Momentum	r_u	τ	Δ
FixMatch	0	5×10^{-4}	0.03	64	0.88	7	0.95	0
Cifar training	1, 4	8×10^{-4}	0.06	30	0.88	9	0.95	0.25
Cifar training	2, 3	8×10^{-4}	0.06	30	0.88	9	0.9	0
Self-training	4	5×10^{-4}	0.03	64	0.88	7	0.95	0.25
SVHN training	1, 4	6×10^{-4}	0.04	32	0.85	7	0.95	0.25
SVHN training	2, 3	6×10^{-4}	0.04	32	0.85	7	0.9	0
Self-training	0	6×10^{-4}	0.04	32	0.85	7	0.95	0.25

Table S1. Hyper-parameter values for each of the various steps in the training.

		BOSS balance method			
WD/LR/BS/r_u	FixMatch	1	2	3	4
$5 \times 10^{-4}/0.03/64/7$	74 ± 5	34 ± 2	44 ± 7	40 ± 2	31.5 ± 0.5
$8 \times 10^{-4}/0.06/30/9$	47 ± 8	93 ± 0.7	90 ± 2	84 ± 13	78 ± 20

Table S2. Test accuracies for class prototype set 2 for two hyper-parameter settings. The hyper-parameters are weight decay (WD), learning rate (LR), batch size (BS), and the ratio of the unlabeled to labeled data (r_u).

dataset can be quickly created from the "discards" when searching for iconic prototypes. A small test dataset is useful for prototype refinement (i.e., deciding which class prototypes to replace) and it provides the practitioner with useful feedback on the system's performance with a little additional effort. But even without any test data, one can utilize the pseudo-labeled class counts to decide which class prototypes should be replaced.

Furthermore, there are several assumptions that might not hold true in a practical setting. First of all, there is an implicit assumption that the unlabeled dataset is class balanced; that is, it contains the same number of samples of each class. In practical situations with large amounts of unlabeled data, this assumption is unlikely to be true. In cases where the number of unlabeled samples belonging to each class can be estimated, it is possible to adapt the class balancing methods. When the number of unlabeled samples belonging to each class is unknown, it is possible to create a small validation set in a similar manner as described above for creating a test set and utilize the validation set as a measure of class balance.

In addition, we also assume in our experiments that all of the unlabeled samples belong to one of the known classes. In practical settings, the unlabeled dataset might contain samples that don't belong to any of the prototype classes. We did not test the situation where we use only a subset of the classes in the training datasets.

2 HYPER-PARAMETERS

For FixMatch we used the default hyper-parameters that were specified in Sohn, et al. Sohn et al. (2020). However, in our initial experiments with the class balance methods, we found that these hyper-parameters performed poorly. Therefore, we used a different set of hyper-parameter values for FixMatch and for the BOSS methods.

Table S1 contains the hyper-parameter values used for the results reported in our paper. Additional hyper-parameter settings that were consistent over all the runs include setting kimgs = 32768 (i.e., the number of training images) and $\lambda_u = 1$ (i.e., the unlabeled loss multiplicative factor). Furthermore, we set the augment input parameter to 'd.d.d', which is the default data augmentation for the labeled and

unlabeled data. Our early experiments with setting the augment input parameter to 'd.d.rac' produces small improvements so we subsequently used the default values. The balance column reflects the class balancing method used (balance = 0 corresponds to FixMatch, which does not use any class balancing method). The remaining columns specify the weight decay, learning rate, batch size, momentum, the ratio of unlabeled to labeled data, confidence threshold, and change in the confidence threshold for minority classes. Details of these last three hyper-parameters are provided in the main text.

Specifically, we found that increasing the ratio of unlabeled to labeled data (from 7 to 9), weight decay (from 5×10^{-4} to 8×10^{-4}) and the learning rates (from 0.03 to 0.06) improved performance. We also found that decreasing the confidence threshold from 0.95 to 0.9 improved performance but for class balancing methods 1 and 4, we left the confidence threshold at 0.95 because the class-based thresholds were lowered by these class balancing methods. We also discovered that a smaller batch-size improved performance and chose a batch size of 30 that was a multiple of the number of classes. Our experiments with momentum found a small improvement with values between 0.85 and 0.9 and settled on using 0.88 for our experiments.

As mentioned above, we tried to use the same hyper-parameters for both FixMatch and for the class balancing methods but this proved to provide an unfair comparison to one or the other. Table S2 illustrates this. This Table provides the averaged test accuracies for class prototype set 2 for the default and another choice of weight decay (WD), learning rate (LR), batch size (BS), and the ratio of the unlabeled to labeled data (r_u). The results for the BOSS methods improve significantly by tuning the hyper-parameters but the performance of FixMatch is reduced substantially. So we used the default set of hyper-parameters for FixMatch and another set of hyper-parameter values for the class balance methods.

3 IMPLEMENTATION DETAILS

In this Section we describe the changes we made to the original FixMatch codes and provide guidance on how to replicate our experiments. This Section relies on the reader being familiar with the TensorFlow version at `https://github.com/google-research/fixmatch` and the PyTorch version located at `https://github.com/CoinCheung/fixmatch`. Our code is available at `https://github.com/lnsmith54/BOSS` to facilitate replication.

Modifications to the original TensorFlow version of the FixMatch code were localized. In the TensorFlow version, the primary changes were made to *fixmatch.py*. This includes the implementation of the four class balancing methods. In support of these methods, the code for computing the number of pseudo-labels in each class was implemented. Also, a few new input parameters were added to this file that are related to the class balancing methods. Specifically, we added the input parameter "balance" to specify the class balancing method (balance=0 acts the same as the original FixMatch code) and "delT" (i.e., Δ) as the amount that balance method 1 can reduce the threshold. Modifications were also made to *cta/lib/train.py* to compute test accuracies for each class, keep track of the best test accuracy, and output the sorted pseudo-labels for the unlabeled training data. In addition, changes to *libml/data.py* and *libml/augment.py* were required in order to accept the new prototype versions of the labeled datasets.

In addition to the code, the TensorFlow FixMatch version required several other steps that are supported by code in the *scripts* folder. Instructions for creating the necessary dataset files are located on the website at `https://github.com/google-research/fixmatch`. These instructions use programs in the scripts folder that needed to be modified in order to create the dataset files needed for the prototype sets and for self-training.

We named the prototype datasets with a 'p' at the end to distinguish them from the original datasets. That is, 'cifar10' became 'cifar10p' and 'svhn' became 'svhnp'. Therefore, it was necessary to create *scripts/cifar10_prototypes.py* and *scripts/svhn_prototypes.py* to generate the labeled training data files. We note that to be consistent with the TensorFlow FixMatch, we used 'seed' as the input parameter to represent different prototype sets. It is also necessary to copy the unlabeled training and labeled training files from the cifar10/svhn file names to the cifar10p/svhnp file names and we provide shell scripts to do so.

Self-training is performed as a separate step from the first training run. The training run will have created three files containing the pseudo-labels for the unlabeled training data sorted from the most confident predictions down. The three files are the pseudo-labels, the confidences, and the true labels (used only for debugging purposes). The programs *scripts/cifar10_iteration.py* and *scripts/svhn_iteration.py* are provided to combine the highest confidence pseudo-labeled examples with the labeled class prototypes and create the necessary files for the self-training run. We provide shell scripts as a template for how this is done. Once these files are created, the self-training iteration can be run.

Most of our experiments were run on a SuperMicro SuperServer with Tesla V100 GPUs. We discovered that it was important to run our experiments on only 1 GPU and all our runs using multiple GPUs performed poorly.

Modifications to the PyTorch version of the FixMatch code were simpler than for the TensorFlow code. However, the execution of this code ran almost three times longer, which greatly reduced the number of experiments we could run due to constraints on computational resources. The primary modifications for class balancing were added to *label_guessor.py*. Secondary modifications were made to the main program in *train.py* to add the class balancing input parameters and arguments for the call to label_guessor. In addition, *cifar.py* was modified to use the class prototypes instead of random examples. It was not necessary to create class prototype files as it was with the TensorFlow version. We did not have sufficient time to test self-training with the PyTorch version.

REFERENCES

Berthelot, D., Carlini, N., Goodfellow, I., Papernot, N., Oliver, A., and Raffel, C. A. (2019). Mixmatch: A holistic approach to semi-supervised learning. In *Advances in Neural Information Processing Systems*. 5050–5060

Gastaldi, X. (2017). Shake-shake regularization. *arXiv preprint arXiv:1705.07485*

Laine, S. and Aila, T. (2017). Temporal ensembling for semi-supervised learning. In *Fifth International Conference on Learning Representations*

Miyato, T., Maeda, S.-i., Ishii, S., and Koyama, M. (2018). Virtual adversarial training: a regularization method for supervised and semi-supervised learning. *IEEE transactions on pattern analysis and machine intelligence*

Sohn, K., Berthelot, D., Carlini, N., Zhang, Z., Zhang, H., Raffel, C. A., et al. (2020). Fixmatch: Simplifying semi-supervised learning with consistency and confidence. *Advances in Neural Information Processing Systems* 33, 596–608

Tarvainen, A. and Valpola, H. (2017). Mean teachers are better role models: Weight-averaged consistency targets improve semi-supervised deep learning results. In *Advances in neural information processing systems*

Verma, V., Lamb, A., Kannala, J., Bengio, Y., and Lopez-Paz, D. (2019). Interpolation consistency training for semi-supervised learning. *arXiv preprint arXiv:1903.03825*

Xie, Q., Dai, Z., Hovy, E., Luong, M.-T., and Le, Q. V. (2019). Unsupervised data augmentation for consistency training

Zhai, X., Oliver, A., Kolesnikov, A., and Beyer, L. (2019). S4l: Self-supervised semi-supervised learning. In *Proceedings of the IEEE international conference on computer vision*. 1476–1485
